# Functional outcome of 2-D- and 3-D-guided corrective forearm osteotomies: a systematic review

**DOI:** 10.1177/17531934231201962

**Published:** 2023-09-25

**Authors:** Anne M. L. Meesters, Nick Assink, Frank F. A. IJpma

**Affiliations:** 1 Department of Trauma Surgery, University Medical Centre Groningen, University of Groningen, Groningen, The Netherlands; 2 3D Lab, University Medical Centre Groningen, University of Groningen, Groningen, The Netherlands

**Keywords:** Systematic review, corrective osteotomy, 3-D, three-dimensional, distal radius, malunion, forearm

## Abstract

We performed a systematic review to compare conventional (2-D) versus 3-D-guided corrective osteotomies regarding intraoperative results, patient-reported outcome measures, range of motion, incidence of complications and pain score. PubMed (MEDLINE), Embase and Cochrane CENTRAL were searched, and 53 articles were included, reporting 1257 patients undergoing forearm corrective osteotomies between 2010 and 2022. 3-D-guided surgery resulted in a greater improvement in median Disabilities of the Arm, Shoulder and Hand (DASH) score (28, SD 7 vs. 35, SD 5) and fewer complications (12% vs. 6%). Pain scores and range of motion were similar between 3-D-guided and conventional surgery. 3-D-guided corrective osteotomy surgery appears to improve patient-reported outcomes and reduce complications compared to conventional methods. However, due to the limited number of comparative studies and the heterogeneity of the studies, a large randomized controlled trial is needed to draw definitive conclusions.

**Level of evidence:** III

## Introduction

A malunion incidence of up to 17% has been reported for distal radial fractures (Bushnell and Bynum, 2007; Katt et al., 2020). In the short term, a malunited forearm fracture can lead to functional impairment, pain, instability and/or aesthetic concerns. In the long term, it can also lead to early-onset osteoarthritis of adjacent joints. Corrective osteotomy surgery can be challenging because the bone is often deformed in multiple planes. Traditionally, osteotomy surgery has been prepared using 2-D radiographs and computed tomography (CT) images, which can lead to unpredictable and inaccurate surgical outcomes (Hoekstra et al., 2016).

Surgeons often cannot properly assess the degree of deformity and accurately correct it with the naked eye. Suboptimal correction in these patients can result in residual functional impairment, pain and joint instability. With the advent of 3-D technologies, corrective osteotomies can now be planned using 3-D visualization and printing tools. In addition, the surgery can be performed using 3-D-printed patient-specific surgical cutting and reposition guides (Lal and Patralekh, 2018; Tack et al., 2016). Various methods and techniques for corrective osteotomies of different deformed bones have been described (Assink et al., 2022a). In addition, 3-D corrective osteotomies for malunited paediatric forearm fractures are already being performed and 3-D osteotomies are a predictor of better functional outcome in children (Roth et al., 2017, 2022a, 2022b). A review by De Muinck Keizer et al. (2017) showed that 3-D-guided corrective osteotomies appear to be a promising technique in the treatment of complex distal radius malunions and may improve both radiographic and functional outcomes. However, this review did not identify any studies comparing the results of 3-D planning techniques with conventional planning methods.

Despite the increasing number of publications on the use of 3-D-guided corrective osteotomies in forearm deformities, a comparison between conventional and 3-D-guided procedures and functional outcome is lacking. This systematic review evaluates functional outcome after 3-D-guided corrective osteotomies compared to conventional corrective osteotomies of the forearm to answer the following research questions: (1) Does the clinical application of 3-D-guided corrective osteotomies improve patient-reported outcome measures compared to conventional surgery for forearm deformities? (2) Does the clinical application of 3-D-guided corrective osteotomies improve the range of motion (ROM), incidence of complications and pain score compared to conventional surgery for forearm deformities? and (3) Does the clinical application of 3-D-guided corrective osteotomies improve intraoperative results in terms of operation time, blood loss and fluoroscopy time compared to conventional surgery for forearm deformities?

## Methods

The Preferred Reporting Items for Systematic Reviews (Moher et al., 2009) were used for this review. The registration number of the review protocol in the International Prospective Register of Systematic Reviews is CRD42022351628.

### Search strategy and study selection

A search string was developed in collaboration with a medical librarian (Online Table S1), and Embase, Cochrane CENTRAL and PubMed including MEDLINE were searched for articles published between 1 January 2010 and 29 July 2022.

Randomized controlled trials (RCTs), cohort studies, case-control studies, cross-sectional studies and case series with at least 10 patients on the treatment of corrective osteotomies of the forearm and wrist were eligible for inclusion. Cadaver studies, paediatric studies (age <16 years), letters to the editor, conference abstracts, systematic reviews, 3-D measurements or analyses, biomechanical studies, statistical shape studies, studies on ulnar shortening or radial lengthening and studies on diseases (e.g. Kienböcks disease, brachial plexus birth palsy/brachial plexus injury, hereditary multiple osteochondromas, Madelung's deformity, cerebral palsy) were excluded along with studies focusing on the operative treatment of synostosis and studies in languages other than English, German or Dutch. Studies reporting on corrective osteotomies in different body regions were only included if the results of corrective osteotomies of the forearm were reported separately (Aibinder et al., 2018; Oka et al., 2019). For these studies, only the results for corrective osteotomies of the forearm and wrist were reported.

Rayyan QCRI (Ouzzani et al., 2016), a web-based sorting tool for systematic literature reviews, was used to screen the articles. All articles were uploaded to Rayyan QCRI and two reviewers (AMLM and NA) independently screened the titles and abstracts of all articles. Differences between the reviewers were resolved in a consensus meeting. The full texts of the remaining articles were then independently screened by the same two reviewers.

### Quality check and data extraction

Methodological quality and risk of bias were assessed independently by two reviewers (AMLM and NA) using the McMaster University Occupational Therapy Evidence-Based Practice Research Group guidelines (Letts et al., 2007). These guidelines consist of questions about the study design and purpose, background literature, sample size, randomization, outcome measures, results, implications and conclusions. Each question was scored 1 point for ‘yes’ and zero points for ‘no’ or NA for ‘not applicable’. The maximum score could be 16 for RCTs, 12 for case series and 14 for other designs. The final scores were expressed as a percentage in the range of 0%–100%, with a higher score indicating higher methodological quality. Scores >90% were considered excellent-quality studies, scores of 75%–90% good-quality studies, scores of 50%–74% moderate-quality studies and <50% poor-quality studies. Where necessary, a consensus meeting was held to resolve disagreements.

### Outcome measurements

The primary outcomes were patient-reported outcome measures using the Disability of the Arm, Shoulder and Hand (DASH) questionnaire (with a score of 0 being perfect and 100 being poor). Secondary outcomes were ROM, visual analogue scale (VAS) score for pain (with zero indicating no pain and 10 indicating the worst pain), incidence of complications (defined as implant failure, loss of the correction, revision corrective osteotomy, tendon injury, nerve injury, infection, malunion or nonunion), operative time, intraoperative blood loss and use of intraoperative fluoroscopy.

### Statistical analysis

The Shapiro–Wilk test showed that the data were not normally distributed. When two or more studies reported an outcome variable, the weighted median and interquartile range (IQR) were calculated for continuous variables, and a percentage was calculated for categorical variables. To assess the differences between conventional and 3-D-guided osteotomies, a chi-square test was performed for categorical variables and a Mann–Whitney U test was performed for continuous variables. A *p*-value <0.05 was considered significant. Authors were successfully contacted to retrieve additional data (age) in one study (Miyake et al., 2012), to only include patients aged >15 years. Moreover, in four articles the mean values were retrieved instead of the median values that were reported (Andreasson et al., 2020a, 2020b; Mulders et al., 2017; Stirling et al., 2020). For one article, additional mean values could not be retrieved (Estermann et al., 2022).

## Results

**Figure 1.**
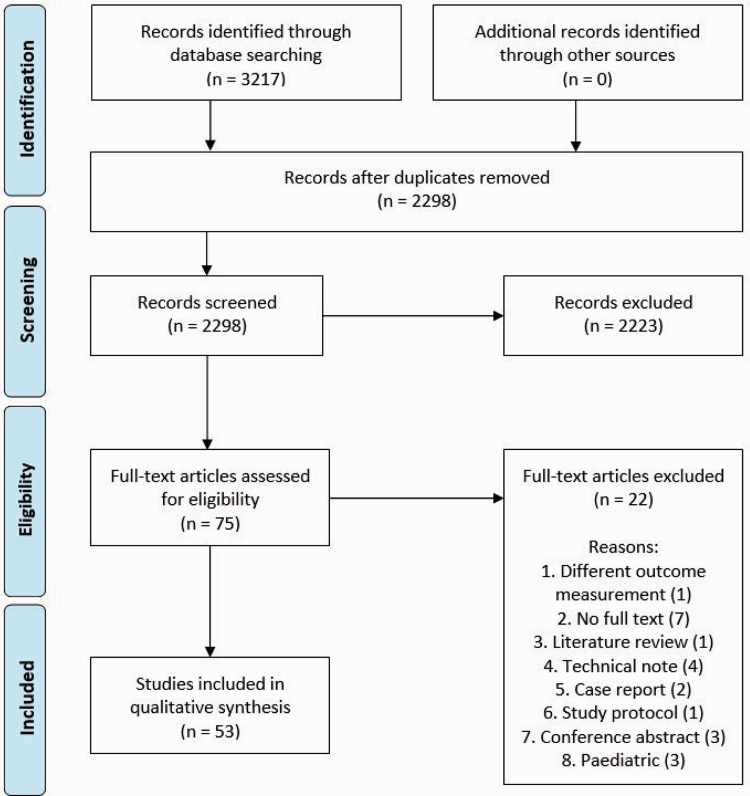
Preferred Reporting Items for Systematic Reviews flow diagram.

In total, 53 studies were included in this review (Figure 1) (Aibinder et al., 2018; Andreasson et al., 2020a, 2020b; Athlani et al., 2020; Bauer et al., 2017; Bhatia et al., 2022; Bilgin and Armangil, 2012; Buijze et al., 2012, 2018; Capo et al., 2010; Cha et al., 2021; Delclaux et al., 2016; Dobbe et al., 2021; Elmi et al., 2014; Estermann et al., 2022; Fok et al., 2015; Gaspar et al., 2017; Gradl et al., 2013; Haghverdian et al., 2019; Hsieh et al., 2010; Huang et al., 2019; Izmalkov et al., 2022; Kiliç et al., 2011; Konul and Krimmer, 2012; Lee et al., 2022; Lozano-Calderón et al., 2010; Mahmoud et al., 2012; Michielsen et al., 2018; Miyake et al., 2011, 2012; Mulders et al., 2017; Oka et al., 2018, 2019; de Oliveira et al., 2012; Opel et al., 2014; Ozasa et al., 2013; Pace et al., 2021; Park et al., 2012; Pecache and Calleja, 2020; Pillukat et al., 2013, 2014, 2018; Robinson et al., 2022; Roner et al., 2020; Rothenfluh et al., 2013; Schurko et al., 2020; Shintani et al., 2018; Singh et al., 2022; Stirling et al., 2020; Tarallo et al., 2014; Tiren and Vos, 2014; Wu, 2017; Zhang et al., 2022). The study characteristics are presented in Online Table S2. Two studies actually compared conventional osteotomies with 3-D-guided osteotomies (Bauer et al., 2017; Buijze et al., 2018). A total of 13 studies involving 254 patients reported on 3-D-guided osteotomies (Athlani et al., 2020; Bauer et al., 2017; Buijze et al., 2018; Dobbe et al., 2021; Estermann et al., 2022; Michielsen et al., 2018; Miyake et al., 2011, 2012; Oka et al., 2018, 2019; Roner et al., 2020; Shintani et al., 2018; Singh et al., 2022). Surgical guides were most often used (Online Fig S1). One study reported the use of patient-specific implants (Dobbe et al., 2021), and one study reported the use of a printed 3-D wedge as a reference during surgery (Shintani et al., 2018). In total, 38 studies representing 1003 patients described osteotomies performed using conventional techniques (2-D radiographs and CT images without cutting and drilling guides).

### Methodological quality assessment

Two randomized controlled trials (Andreasson et al., 2020b; Buijze et al., 2018), two case-control studies (Aibinder et al., 2018; Bauer et al., 2017), 12 cohort studies (Athlani et al., 2020; Bhatia et al., 2022; Dobbe et al., 2021; Kiliç et al., 2011; Mahmoud et al., 2012; Michielsen et al., 2018; Miyake et al., 2012; Oka et al., 2019; Pillukat et al., 2013, 2018, 2014; Tarallo et al., 2014) and 37 case studies (Andreasson et al., 2020a; Bilgin and Armangil, 2012; Buijze et al., 2012; Capo et al., 2010; Cha et al., 2021; Delclaux et al., 2016; Elmi et al., 2014; Estermann et al., 2022; Fok et al., 2015; Gaspar et al., 2017; Gradl et al., 2013; Haghverdian et al., 2019; Hsieh et al., 2010; Huang et al., 2019; Izmalkov et al., 2022; Konul and Krimmer, 2012; Lee et al., 2022; Lozano-Calderón et al., 2010; Miyake et al., 2011; Mulders et al., 2017; Oka et al., 2018; de Oliveira et al., 2012; Opel et al., 2014; Ozasa et al., 2013; Pace et al., 2021; Park et al., 2012; Pecache and Calleja, 2020; Robinson et al., 2022; Roner et al., 2020; Rothenfluh et al., 2013; Schurko et al., 2020; Shintani et al., 2018; Singh et al., 2022; Stirling et al., 2020; Tiren and Vos, 2014; Wu, 2017; Zhang et al., 2022) were included. Nine studies were of excellent quality, 18 studies were of good quality, 21 studies were of moderate quality and five studies were of poor quality (Online Table S3–S6). The median McMaster score was 75% (IQR 58%–83%).

### Patient-reported outcome measurements

Preoperative mean QuickDASH score was reported in 25 conventional (2-D) studies, and the postoperative mean QuickDASH score was reported in 38 conventional studies (Table 1). The preoperative mean DASH score was reported in five 3-D-guided studies, and the postoperative mean DASH score in seven 3-D-guided studies (Table 1). An improvement in postoperative DASH score compared to preoperative DASH score was reported in six studies (Table 1).

**Table 1.** Conventional and 3-D-guided pre- and postoperative QuickDASH and VAS pain scores.

Measurement	Conventional		3-D-guided		*p*-value
	N	Weighted median (IQR)	N	Weighted median (IQR)	
Preoperative QuickDASH	422	45.0 (36.3–55.0)	77	46.0 (46.0–51.8)	0.188
Postoperative QuickDASH	660	17.4 (15.0–20.2)	106	17.0 (16.1–18.8)	0.331
Improvement DASH	103	25.1 (25.1–28.5)	36	35.6 (33.2–38.1)	**<0.001**
Preoperative VAS	372	5.6 (4.1–6.3)	87	5.0 (4.9–5.0)	**0.004**
Postoperative VAS	412	2.0 (1.0–2.2)	102	2.0 (0.8–2.0)	0.374

Values in bold indicate a significant *p*-value.

DASH: Disabilities of the Arm, Shoulder and Hand; IQR: interquartile range; N: number of patients in all studies; VAS: visual analogue scale.

### Range of motion

The preoperative ROM was reported in 24 trials, four of which were 3-D-guided osteotomy trials. The postoperative ROM was reported in 36 studies, six of which were 3-D-guided osteotomy studies. The postoperative weighted mean ROM improved in both the conventional and the 3-D-guided osteotomy groups (Table 2). Postoperative wrist flexion, pronation, supination, radial deviation and ulnar deviation were better in the 3-D-guided group compared to the conventional group.

**Table 2.** Pre- and postoperative range of motion measurements in the conventional osteotomy versus 3-D-guided osteotomy studies.

Measurement	Conventional		3-D-guided		*p*-value
	N	Weighted median (°)	N	Weighted median (°)	
Preoperative wrist flexion	382	40 (27–45)	37	33 (33–39)	0.342
Postoperative wrist flexion	538	59 (53–60)	72	54 (50–61)	**0.002**
Preoperative wrist extension	382	45 (38–66)	37	48 (48–63)	0.125
Postoperative wrist extension	538	63 (56–70)	72	64 (61–66)	0.798
Preoperative pronation	341	63 (57–74)	53	71 (59–77)	**<0.001**
Postoperative pronation	497	77 (70–81)	88	81 (77–84)	**<0.001**
Preoperative supination	341	57 (41–68)	53	62 (45–72)	**0.002**
Postoperative supination	497	77 (70–80)	88	81 (78–84)	**<0.001**
Preoperative radial deviation	252	14 (13–21)	N/A	N/A	N/A
Postoperative radial deviation	395	17 (13–19)	35	26 (19–26)	**<0.001**
Preoperative ulnar deviation	252	19 (17–27)	N/A	N/A	N/A
Postoperative ulnar deviation	395	27 (23–34)	35	40 (33–40)	**<0.001**

Values in parentheses are IQR. Values in bold indicate a significant *p*-value.

IQR: interquartile range; N: number of patients; N/A, not applicable.

Some trials only reported improvements in the ROM, but the results were too heterogeneous to be included (Bauer et al., 2017; Michielsen et al., 2018; Pace et al., 2021; Roner et al., 2020).

### Visual analogue scale (VAS) pain

The preoperative VAS score was reported in 20 conventional studies and the postoperative VAS score in 23 conventional studies (Table 1). The preoperative VAS score was reported in six 3-D-guided osteotomy studies and the postoperative VAS score in seven 3-D-guided osteotomy studies (Table 1).

### Complications

Complications were reported in 45 studies (Online Table S2). In total, 34 were conventional studies including 856 patients with 100 (11.7%) complications. Thirteen 3-D-guided osteotomy studies reported 254 patients with 16 (6.3%) complications, a significantly lower incidence compared to the conventional studies (*p* < 0.001). The different types of complications and percentages for conventional and 3-D-guided osteotomies are presented in Online Table S7.

### Operation time, intraoperative blood loss and intraoperative fluoroscopy usage

Only two studies reported operative time (Bauer et al., 2017; Buijze et al., 2018). Bauer et al. (2017) reported a significantly shorter operative time in the 3-D-guided osteotomy group compared to the conventional osteotomy group (140 minutes [SD 37] vs. 108 minutes [SD 26]; *p < *0.05). Buijze et al. (2018) reported a slightly shorter operative time for the 3-D-guided group compared to the conventional group (91 minutes [SD 32] vs. 97 minutes [SD 34]; *p = *0.58).They also reported a significantly shorter intraoperative fluoroscopy time for 3-D-guided osteotomies compared to conventional osteotomies (58 seconds [SD 38) vs. 140 seconds [SD 101]; *p* = 0.01) (Buijze et al., 2018). No trials reported intraoperative blood loss.

## Discussion

In recent years, the use of 3-D technology to guide corrective osteotomy surgery has found its way to the clinical treatment of malunited forearm bones. 3-D-guided corrective osteotomies in the treatment of complex distal radial fractures lead to satisfactory radiographic and functional outcome (De Muinck Keizer et al., 2017). This review of 53 studies representing 1003 conventional and 254 3-D-guided patients indicates that the use of 3-D guidance during corrective osteotomy surgery leads to a higher improvement in DASH score after surgery and fewer complications compared to conventional methods.

No difference was found between both the preoperative and postoperative scores when comparing the weighted mean of the DASH and VAS scores of the conventionally and 3-D-guided treated patients. However, the number of studies only reporting on postoperative functional outcome was greater than the studies that also included the preoperative outcome. Since the preoperative functional outcome is essential to fully assess the effect of the intervention, it might be a fairer comparison to assess the gained improvement in DASH score rather than the score after the surgery. Studies that reported the gained improvement in DASH score did show a significantly higher gained DASH score in the patients treated with the 3-D-guided method (an increase of 35 [SD 5] compared to an increase of 28 [SD 7] for conventional corrective osteotomies). The postoperative DASH values found within our review were similar to the studies performed by Prommersberger et al. (2012) and Wada et al. (2011) (postoperative DASH scores of 11 and 13, respectively). These studies provided an overview of functional outcomes after conventional treatment. Our review adds 3-D-guided osteotomy results and shows that 3-D-guided osteotomies lead to a higher improvement in functional outcome compared to conventional osteotomies.

For ROM, the 3-D-guided method resulted in a significantly better postoperative wrist flexion, pronation, supination and ulnar and radial deviation. However, it would be more helpful to assess a gained ROM rather than only the postoperative ROM. Yet, the difference between pre- and postoperative ROM was only available in four studies (Bauer et al., 2017; Michielsen et al., 2018; Pace et al., 2021; Roner et al., 2020). Bauer et al. (2017) reported a gain in pro-/supination of 41° (SD 39°) in the conventional group compared to a gain of 43° (SD 27°) in the 3-D-guided group. Moreover, they reported a gain in flexion/extension of 28° (SD 41°) in the conventional group and 25° (SD 36°) in the 3-D-guided group. This indicates that 3-D-guided osteotomies yield similar results compared to conventional osteotomies, although usually more severe malunions were treated with the 3-D-guided method. Other studies reported similar pre- and postoperative ROM compared to the results of our review (De Muinck Keizer et al., 2017; Prommersberger et al., 2012; Schweizer et al., 2013). Byrne et al. (2017) reported a higher ROM, but they only included paediatric patients. Overall, in most studies as well as in our review, 3-D-guided surgery resulted in a better improvement of ROM than conventional surgery.

Functional outcome in terms of operating time, intraoperative blood loss and use of intraoperative fluoroscopy could not be thoroughly assessed because only two trials reported some of these variables. However, these two studies compared 3-D-guided osteotomies to conventional osteotomies and found a significantly shorter operating time (Bauer et al., 2017) and less use of fluoroscopy (Buijze et al., 2018). This is in line with previous reviews of 3-D-guided fracture surgery, which also showed shorter operating times and less use of fluoroscopy for 3-D-guided patients (Assink et al., 2022b; Meesters et al., 2021). Shorter operating time and less fluoroscopy use could be explained by the extensive preoperative planning for 3-D-guided osteotomies. Therefore, it is recommended to include operative time, intraoperative blood loss and intraoperative fluoroscopy use in future research. Conversely, the additional time and cost of preoperative planning should also be considered.

A limitation of our review is the heterogenous patient population in the included studies. Moreover, conventional correction methods included anterior and dorsal surgical approaches, treatment with and without graft, and intra- and extra-articular corrections, which make comparisons more difficult. In addition, three different 3-D-guided correction methods were identified in this review. For practical reasons, all patients treated with any conventional method were included in the conventional group, and all patients with a 3-D-guided method in the 3-D group. Therefore, the results of this review should be interpreted in face of this limitation.

Despite the heterogenous character of the forearm deformities overall, the 3-D method is at least non-inferior. One should take into account that two conventional (Pillukat et al., 2014, 2013) and two 3-D-guided studies (Roner et al., 2020; Singh et al., 2022) have a partly overlapping inclusion period, but it is unclear whether this means that the same patients (17 conventional and 15 3-D-guided) were included twice. Another limitation is that the majority of the included studies were non-comparative studies and described only either the conventional or the 3-D-guided corrective osteotomies. Comparison between these studies is complicated due to differences in patient characteristics and outcome measures. Patient-reported outcomes varied highly between studies with regard to the follow-up moment (i.e. preoperative, postoperative or improvement) and timeframe (a few months to several years). Only one RCT (Buijze et al., 2018) and one case-control study (Bauer et al., 2017) were included in which conventional and 3-D-guided osteotomies were directly compared. Both studies, however, lacked sufficient power and emphasized the need for a large trial to clearly define the clinical benefits of the 3-D-guided technique.

In light of the additional costs in terms of preparation time and production costs of the 3-D-guided workflow, the potential benefits should be further investigated and balanced with the potential benefits, including lower costs of productivity loss (return to work) and lower direct medical costs (consumption of healthcare) before widespread use.

## Supplemental Material

sj-zip-1-jhs-10.1177_17531934231201962 - Supplemental material for Functional outcome of 2-D- and 3-D-guided corrective forearm osteotomies: a systematic review Supplemental material, sj-zip-1-jhs-10.1177_17531934231201962 for Functional outcome of 2-D- and 3-D-guided corrective forearm osteotomies: a systematic review by Anne M. L. Meesters, Nick Assink and Frank F. A. IJpma in Journal of Hand Surgery (European Volume)

sj-pdf-2-jhs-10.1177_17531934231201962 - Supplemental material for Functional outcome of 2-D- and 3-D-guided corrective forearm osteotomies: a systematic review Supplemental material, sj-pdf-2-jhs-10.1177_17531934231201962 for Functional outcome of 2-D- and 3-D-guided corrective forearm osteotomies: a systematic review by Anne M. L. Meesters, Nick Assink and Frank F. A. IJpma in Journal of Hand Surgery (European Volume)

sj-pdf-3-jhs-10.1177_17531934231201962 - Supplemental material for Functional outcome of 2-D- and 3-D-guided corrective forearm osteotomies: a systematic review Supplemental material, sj-pdf-3-jhs-10.1177_17531934231201962 for Functional outcome of 2-D- and 3-D-guided corrective forearm osteotomies: a systematic review by Anne M. L. Meesters, Nick Assink and Frank F. A. IJpma in Journal of Hand Surgery (European Volume)

sj-pdf-4-jhs-10.1177_17531934231201962 - Supplemental material for Functional outcome of 2-D- and 3-D-guided corrective forearm osteotomies: a systematic review Supplemental material, sj-pdf-4-jhs-10.1177_17531934231201962 for Functional outcome of 2-D- and 3-D-guided corrective forearm osteotomies: a systematic review by Anne M. L. Meesters, Nick Assink and Frank F. A. IJpma in Journal of Hand Surgery (European Volume)

sj-pdf-5-jhs-10.1177_17531934231201962 - Supplemental material for Functional outcome of 2-D- and 3-D-guided corrective forearm osteotomies: a systematic review Supplemental material, sj-pdf-5-jhs-10.1177_17531934231201962 for Functional outcome of 2-D- and 3-D-guided corrective forearm osteotomies: a systematic review by Anne M. L. Meesters, Nick Assink and Frank F. A. IJpma in Journal of Hand Surgery (European Volume)

sj-pdf-6-jhs-10.1177_17531934231201962 - Supplemental material for Functional outcome of 2-D- and 3-D-guided corrective forearm osteotomies: a systematic review Supplemental material, sj-pdf-6-jhs-10.1177_17531934231201962 for Functional outcome of 2-D- and 3-D-guided corrective forearm osteotomies: a systematic review by Anne M. L. Meesters, , Nick Assink and Frank F. A. IJpma in Journal of Hand Surgery (European Volume)

sj-pdf-7-jhs-10.1177_17531934231201962 - Supplemental material for Functional outcome of 2-D- and 3-D-guided corrective forearm osteotomies: a systematic review Supplemental material, sj-pdf-7-jhs-10.1177_17531934231201962 for Functional outcome of 2-D- and 3-D-guided corrective forearm osteotomies: a systematic review by Anne M. L. Meesters, Nick Assink and Frank F. A. IJpma in Journal of Hand Surgery (European Volume)

sj-pdf-8-jhs-10.1177_17531934231201962 - Supplemental material for Functional outcome of 2-D- and 3-D-guided corrective forearm osteotomies: a systematic review Supplemental material, sj-pdf-8-jhs-10.1177_17531934231201962 for Functional outcome of 2-D- and 3-D-guided corrective forearm osteotomies: a systematic review by Anne M. L. Meesters, Nick Assink and Frank F. A. IJpma in Journal of Hand Surgery (European Volume)
