# Supplementary material for: Functional outcome of 2-D- and 3-D-guided corrective forearm osteotomies: a systematic review
Source: J Hand Surg Eur Vol. 2023 Sep 25;49(7):843–51. doi: 10.1177/17531934231201962 (PMC11264531; doi:10.1177/17531934231201962)
Supplement: sj-pdf-2-jhs-10.1177_17531934231201962 - Supplemental material for Functional outcome of 2-D- and 3-D-guided corrective forearm osteotomies: a systematic review [file sj-pdf-2-jhs-10.1177_17531934231201962.pdf]

Online Table S1: Search string.

Database	Search string
PubMed including MEDLINE	(Radius*[tiab] OR ‘Radius’ [Mesh] OR Forearm*[tiab] OR ‘Forearm’ [Mesh]) AND (malunion[tiab] OR deformity*[tiab] OR ‘Osteotomy’ [Mesh] OR osteotomy*[tiab] OR osteotomies*[tiab]) AND ‘2010/01/01’ [PDat]: ‘3000/12/31’ [PDat]
Embase	('radius'/exp OR radius*:ti,ab OR ‘forearm’/exp OR forearm*:ti,ab) AND (malunion*:ti,ab OR deformity*:ti,ab OR ‘Osteotomy’/exp OR osteotomy*:ti,ab OR osteotomies*:ti,ab) AND [embase]/lim AND [2010-2022]/py
Cochrane CENTRAL	((radius):ti,ab,kw OR (forearm):ti,ab,kw) AND ((malunion):ti,ab,kw OR (deformity):ti,ab,kw OR (osteotomy):ti,ab,kw OR (osteotomies):ti,ab,kw)" with Publication Year from 2010 to 2022, in Trials
