# Supplementary material for: Functional outcome of 2-D- and 3-D-guided corrective forearm osteotomies: a systematic review
Source: J Hand Surg Eur Vol. 2023 Sep 25;49(7):843–51. doi: 10.1177/17531934231201962 (PMC11264531; doi:10.1177/17531934231201962)
Supplement: sj-pdf-3-jhs-10.1177_17531934231201962 - Supplemental material for Functional outcome of 2-D- and 3-D-guided corrective forearm osteotomies: a systematic review [file sj-pdf-3-jhs-10.1177_17531934231201962.pdf]

Online Table S2: Study characteristics. Quality was assessed by the McMaster criteria.

| Study | Country | Design | N | Period | Outcome measurements | 2D or 3D | Quality |
|---|---|---|---|---|---|---|---|
| Aibinder et al. 2018 (Aibinder et al., 2018) | NS | Case control | 11 | NS | ROM, VAS | 2D | 57% |
| Andreasson et al. 2020 (Andreasson et al., 2020a) | Sweden | Case study | 37 | 2007 – 2014 | PROMs, ROM, complications | 2D | 75% |
| Andreasson er al. 2020 (Andreasson et al., 2020b) | Sweden | RCT | 38 | 2014-2018 | PROMs, ROM, complications | 2D | 94% |
| Athlani et al. 2020 (Athlani et al., 2020) | France | Cohort study | 16 | 2016 – 2019 | PROMs, VAS, complications | 3D | 67% |
| Bauer et al. 2017 (Bauer et al., 2017) | Switzerland | Case control study | 31 2D 25 3D | 2003 – 2015 | ROM, complications | Both | 71% |
| Bhatia et al. 2022 (Bhatia et al., 2022) | India | Cohort study | 15 | NS | PROMs, ROM, VAS, complications | 2D | 58% |
| Bilgin et al. 2012 (Bilgin and Armangil, 2012) | Turkey | Case study | 11 | 2002 – 2009 | Complications | 2D | 58% |
| Buijze et al. 2012 (Buijze et al., 2012) | NS | Case study | 18 | 1998 – 2009 | PROMs, ROM, VAS, complications | 2D | 67% |
| Buijze et al. 2018 (Buijze et al., 2018) | USA & Europe | RCT | 17 2D 20 3D | 2010 – 2015 | PROMs, ROM, VAS, complications | Both | 100% |
| Capo et al. 2010 (Capo et al., 2010) | USA | Case study | 13 | 2004 – 2007 | PROMs, ROM, complications | 2D | 67% |
| Cha et al. 2021 (Cha et al., 2021) | South-Korea | Case study | 13 | 2000 – 2018 | PROMs, VAS, complications | 2D | 75% |
| Delclaux et al. 2016 (Delclaux et al., 2016) | France | Case study | 12 | 2005 – 2012 | PROMs, ROM, VAS, complications | 2D | 42% |
| De Oliveira et al. 2012 (de Oliveira et al., 2012) | Brazil | Case study | 20 | 2002 – 2009 | ROM, VAS, complications | 2D | 50% |
| Dobbe et al. 2021 (Dobbe et al., 2021) | The Netherlands | Cohort study | 10 | 2017 – 2019 | VAS, complications | 3D | 83% |

| | | | | | | | |
|---|---|---|---|---|---|---|---|
| Estermann et al. 2022 (Estermann et al., 2022) | Switzerland | Case study | 14 | 2012 – 2020 | PROMs, ROM, VAS, complications | 3D | 67% |
| Elmi et al. 2014 (Elmi et al., 2014) | Iran | Case study | 14 | 2005 – 2011 | ROM, VAS, complications | 2D | 42% |
| Fok et al. 2015 (Fok et al., 2015) | Switzerland | Case study | 12 | 2003 – 2011 | ROM, complications | 2D | 33% |
| Gaspar et al. 2017 (Gaspar et al., 2017) | NS | Case study | 39 | 2008 – 2014 | PROMs, VAS, complications | 2D | 67% |
| Gradl et al. 2013 (Gradl et al., 2013) | NS | Case study | 18 | 1999 - 2007 | PROMs, VAS, complications | 2D | 67% |
| Haghverdian et al. 2019 (Haghverdian et al., 2019) | USA | Case study | 60 | 2007 – 2015 | Complications | 2D | 92% |
| Hsieh et al. 2010 (Hsieh et al., 2010) | China | Case study | 12 | 2002 – 2007 | PROMs | 2D | 50% |
| Huang et al. 2019 (Huang et al., 2019) | Taiwan | Case study | 10 | 2014 – 2016 | PROMs, VAS, complications | 2D | 42% |
| Izmalkov et al. 2022 (Izmalkov et al., 2022) | Russia | Case study | 32 | 2008 – 2018 | PROMs, VAS, complications | 2D | 83% |
| Kilic et al. 2011 (Kiliç et al., 2011) | Turkey | Cohort study | 17 | 2005 – 2009 | PROMs, ROM, complications | 2D | 92% |
| Konul et al. 2012 (Konul and Krimmer, 2012) | NS | Case study | 19 | 2005 – 2008 | PROMs, ROM | 2D | 92% |
| Lee et al. 2022 (Lee et al., 2022) | South-Korea | Case study | 22 | 2006 – 2018 | PROMs, complications | 2D | 83% |
| Lozano et al. 2010 (Lozano-Calderón et al., 2010) | The Netherlands & USA | Case study | 22 | 1980 – 2000 | PROMs, ROM | 2D | 100% |
| Mahmoud et al. 2012 (Mahmoud et al., 2012) | Egypt | Cohort study | 19 | 2004 – 2009 | PROMs, ROM, VAS, complications | 2D | 83% |
| Michielsen et al. 2018 (Michielsen et al., 2018) | Belgium | Cohort study | 30 | 2009 – 2014 | PROMs, ROM, VAS, complications | 3D | 50% |

| | | | | | | | |
|---|---|---|---|---|---|---|---|
| Miyake et al. 2011 (Miyake et al., 2011) | Japan | Case study | 10 | 2008 – 2009 | ROM, complications | 3D | 75% |
| Miyake et al. 2012 (Miyake et al., 2012) | Japan | Cohort study | 20; 13 ≥ 16y | 2002 – 2009 | ROM, complications | 3D | 58% |
| Mulders et al. 2017 (Mulders et al., 2017) | The Netherlands | Case study | 48 | 2009 – 2016 | PROMs, ROM, VAS, complications | 2D | 83% |
| Oka et al. 2017 (Oka et al., 2018) | Japan | Case study | 28 | 2003 – 2013 | Complications | 3D | 92% |
| Oka et al. 2019 (Oka et al., 2019) | Japan | Cohort study | 16; 11 forearm | 2015 – 2017 | PROMs, ROM, VAS, complications | 3D | 83% |
| Opel et al. 2013 (Opel et al., 2014) | UK | Case study | 20 | NS | PROMs, ROM, complications | 2D | 58% |
| Ozasa et al. 2013 (Ozasa et al., 2013) | Japan | Case study | 19 | 2000 – 2010 | PROMs | 2D | 83% |
| Pace et al. 2021 (Pace et al., 2021) | Italy | Case study | 32 | NS | PROMs, ROM, VAS, complications | 2D | 83% |
| Park et al. 2012 (Park et al., 2012) | South-Korea | Case study | 14 | 2001 – 2008 | ROM, VAS, complications | 2D | 83% |
| Pecache et al. 2021 (Pecache and Calleja, 2020) | Philippines | Case study | 13 | NS | PROMs | 2D | 42% |
| Pillukat et al. 2013 (Pillukat et al., 2013) | Germany | Cohort study | 34 | 1999 – 2004 | PROMs, ROM, VAS | 2D | 67% |
| Pillukat et al. 2014 (Pillukat et al., 2014) | Germany | Cohort study | 17 | 1992 – 2003 | PROMs, ROM, VAS | 2D | 67% |
| Pillukat et al. 2018 (Pillukat et al., 2018) | Germany | Cohort study | 19; 15 with follow-up | 2013 – 2016 | PROMs, ROM, VAS, complications | 2D | 75% |
| Robinson et al. 2021 (Robinson et al., 2022) | USA | Case study | 12 | 2016 – 2019 | ROM, complications | 2D | 50% |
| Roner et al. 2020 (Roner et al., 2020) | Switzerland | Case study | 37 | 2008 – 2016 | ROM, complications | 3D | 50% |

| | | | | | | | |
|---|---|---|---|---|---|---|---|
| Rothenfluh et al. 2013 (Rothenfluh et al., 2013) | Switzerland | Case study | 22 | 1999 – 2009 | PROMs, ROM, VAS, complications | 2D | 67% |
| Schurko et al. 2020 (Schurko et al., 2020) | USA | Case study | 53 | 2005 – 2017 | PROMs, complications | 2D | 100% |
| Shintani et al. 2018 (Shintani et al., 2018) | Japan | Case study | 19 | NS | ROM, complications | 3D | 50% |
| Singh et al. 2022 (Singh et al., 2022) | Switzerland | Case study | 15 | 2008 – 2015 | PROMs, ROM, VAS, complications | 3D | 75% |
| Stirling et al. 2020 (Stirling et al., 2020) | UK | Case study | 89 | 2010 – 2018 | PROMs, complications | 2D | 83% |
| Tarallo et al. 2014 (Tarallo et al., 2014) | Italy | Cohort study | 20 | 2005 – 2012 | PROMs, ROM, VAS, complications | 2D | 75% |
| Tiren et al. 2014 (Tiren and Vos, 2014) | The Netherlands | Case study | 11 | 2009 – 2011 | PROMs, ROM, complications | 2D | 92% |
| Wu et al. 2017 (Wu, 2017) | Taiwan | Case study | 41 | 2005 – 2014 | PROMs, ROM, complications | 2D | 75% |
| Zhang et al. 2022 (Zhang et al., 2022) | China | Case study | 17 | 2016 – 2018 | PROMs, complications | 2D | 83% |

RCT: Randomized Controlled Trial; PROMs: Patient-Reported Outcome Measurements; ROM: Range of motion; VAS: Visual analogue scale for pain; NS: Not stated
