# Supplementary material for: Functional outcome of 2-D- and 3-D-guided corrective forearm osteotomies: a systematic review
Source: J Hand Surg Eur Vol. 2023 Sep 25;49(7):843–51. doi: 10.1177/17531934231201962 (PMC11264531; doi:10.1177/17531934231201962)
Supplement: sj-pdf-4-jhs-10.1177_17531934231201962 - Supplemental material for Functional outcome of 2-D- and 3-D-guided corrective forearm osteotomies: a systematic review [file sj-pdf-4-jhs-10.1177_17531934231201962.pdf]

Online Table S3: Quality assessment according to the McMaster score, part one.

Categories	Aibinder, 2018	Andreasson, 2020[1]	Andreasson, 2020[2]	Athlani, 2020	Bauer, 2017	Bhatia, 2020	Bilgin, 2012	Buijze, 2012	Buijze, 2018	Capo, 2010	Cha, 2021	Delclaux, 2016	De Oliveira, 2012	Dobbe, 2021
1. Study purpose														
Was the study question clearly stated?	1	1	1	1	1	0	1	1	1	1	0	1	0	1
2. Literature review														
Was relevant background literature reviewed?	1	1	1	0	0	1	0	0	1	1	1	0	1	1
3. Study design	CC	CS	RCT	C	CC	C	CS	CS	RCT	CS	CS	CS	CS	C
4. Sample														
Was the sample described in detail?	1	1	1	0	1	1	1	1	1	0	1	0	1	1
Was the sample justified?	0	0	0	0	1	0	1	1	1	1	1	1	1	1
Were the groups randomized?	0	0	1	0	0	0	0	0	1	0	0	0	0	0
Was randomizing appropriate done?	N/A	N/A	1	N/A	N/A	N/A	N/A	N/A	1	N/A	N/A	N/A	N/A	N/A
5. Outcomes														
Were the outcome measures reliable?	0	1	1	1	0	1	1	1	1	1	1	1	1	1
Were the outcome measures valid?	1	1	1	1	1	1	1	1	1	1	1	1	0	1
6. Intervention														
Intervention was described in detail?	1	1	1	1	1	1	0	1	1	1	1	1	1	1
Contamination was avoided?	1	N/A	1	N/A	1	N/A	N/A	N/A	1	N/A	N/A	N/A	N/A	N/A
Cointervention was avoided?	1	N/A	1	N/A	1	N/A	N/A	N/A	1	N/A	N/A	N/A	N/A	N/A
7. Results														
Results were reported in terms of statistical significance?	1	1	1	1	1	1	0	1	1	0	1	0	1	1
Were the analysis method/s appropriate?	0	1	1	1	1	0	0	1	1	0	1	0	0	1

Clinical importance was reported?	0	0	1	1	0	0	0	0	1	1	0	0	0	1
Drop-outs were reported?	0	1	1	1	0	0	1	0	1	1	1	0	0	0
8. Conclusion														
Conclusions were appropriate given study methods and results?	0	0	1	0	1	1	1	0	1	0	0	0	0	0
Total	8	9	15	8	10	7	7	8	16	8	9	5	6	10
%	57	75	94	67	71	58	58	67	100	67	75	42	50	83

Yes = 1 point; No = 0 points; CC = Case Control study; CR = Case study; RCT = Randomised Controlled Trial; C = Cohort study; N/A = Not applicable

1: Andreasson et al. Long-term outcomes of corrective osteotomy for malunited fractures of the distal radius. J Plast Surg Hand Surg. 2020, 54: 94–100.

2: Andreasson et al. Functional outcome after corrective osteotomy for malunion of the distal radius: a randomised, controlled, double-blind trial. Int Orthop. 2020, 44: 1353–65.
