# Supplementary material for: Functional outcome of 2-D- and 3-D-guided corrective forearm osteotomies: a systematic review
Source: J Hand Surg Eur Vol. 2023 Sep 25;49(7):843–51. doi: 10.1177/17531934231201962 (PMC11264531; doi:10.1177/17531934231201962)
Supplement: sj-pdf-5-jhs-10.1177_17531934231201962 - Supplemental material for Functional outcome of 2-D- and 3-D-guided corrective forearm osteotomies: a systematic review [file sj-pdf-5-jhs-10.1177_17531934231201962.pdf]

Online Table S4: Quality assessment according to the McMaster score, part two.

Categories	Estermann, 2022	Elmi, 2014	Fok, 2015	Gaspar, 2017	Gradl, 2013	Hsieh, 2010	Haghverdian, 2019	Huang, 2019	Izmalkov, 2022	Kilic, 2011	Konul, 2012	Lee, 2022	Lozano, 2010	Mahmoud 2012
1. Study purpose														
Was the study question clearly stated?	1	1	0	1	1	0	1	0	1	1	0	0	1	1
2. Literature review														
Was relevant background literature reviewed?	1	0	0	0	0	0	1	0	0	1	1	1	1	0
3. Study design	CS	CS	CS	CS	CS	CS	CS	CS	CS	C	CS	CS	CS	C
4. Sample														
Was the sample described in detail?	0	0	0	1	1	1	1	0	1	1	1	1	1	1
Was the sample justified?	1	1	1	1	1	1	1	0	1	1	1	1	1	1
Were the groups randomized?	0	0	0	0	0	0	0	0	0	0	0	0	0	0
Was randomizing appropriate done?	N/A	N/A	N/A	N/A	N/A	N/A	N/A	N/A	N/A	N/A	N/A	N/A	N/A	N/A
5. Outcomes														
Were the outcome measures reliable?	1	0	0	1	1	1	1	1	1	1	1	1	1	1
Were the outcome measures valid?	1	0	1	1	1	1	1	1	1	1	1	0	1	1
6. Intervention														
Intervention was described in detail?	1	1	1	1	1	1	1	1	1	1	1	1	1	1
Contamination was avoided?	N/A	N/A	N/A	N/A	N/A	N/A	N/A	N/A	N/A	N/A	N/A	N/A	N/A	N/A
Cointervention was avoided?	N/A	N/A	N/A	N/A	N/A	N/A	N/A	N/A	N/A	N/A	N/A	N/A	N/A	N/A
7. Results														
Results were reported in terms of statistical significance?	1	1	0	1	1	0	1	1	1	1	1	1	1	1

Were the analysis method/s appropriate?	1	1	1	1	1	1	1	1	1	1	1	1	1	1
Clinical importance was reported?	0	0	0	0	0	0	1	0	0	1	1	1	1	1
Drop-outs were reported?	0	0	0	0	0	0	1	0	1	0	1	1	1	1
8. Conclusion														
Conclusions were appropriate given study methods and results?	0	0	0	0	0	0	0	0	1	1	1	1	1	0
Total	8	5	4	8	8	6	11	5	10	11	11	10	12	10
%	67	42	33	67	67	50	92	42	83	92	92	83	100	83

Yes = 1 point; No = 0 points; CC = Case Control study; CR = Case study; RCT = Randomised Controlled Trial; C = Cohort study; N/A = Not applicable
