# Supplementary material for: Functional outcome of 2-D- and 3-D-guided corrective forearm osteotomies: a systematic review
Source: J Hand Surg Eur Vol. 2023 Sep 25;49(7):843–51. doi: 10.1177/17531934231201962 (PMC11264531; doi:10.1177/17531934231201962)
Supplement: sj-pdf-6-jhs-10.1177_17531934231201962 - Supplemental material for Functional outcome of 2-D- and 3-D-guided corrective forearm osteotomies: a systematic review [file sj-pdf-6-jhs-10.1177_17531934231201962.pdf]

Online Table S5: Quality assessment according to the McMaster score, part three.

Categories	Michielsen, 2018	Miyake, 2011	Miyake, 2012	Mulders, 2017	Oka, 2017	Oka, 2019	Opel, 2014	Ozasa, 2013	Pace, 2012	Park, 2012	Pecache, 2021	Pillukat, 2013	Pillukat, 2014	Pillukat, 2018
1. Study purpose														
Was the study question clearly stated?	0	1	0	1	1	1	1	0	1	1	0	1	1	1
2. Literature review														
Was relevant background literature reviewed?	0	1	0	1	0	0	1	1	1	0	1	0	0	1
3. Study design	C	CS	C	CS	CS	C	CS	CS	CS	CS	CS	C	C	C
4. Sample														
Was the sample described in detail?	1	1	1	1	1	1	1	1	1	1	0	1	1	1
Was the sample justified?	1	1	1	1	1	1	0	1	0	1	0	1	1	0
Were the groups randomized?	0	0	0	0	0	0	0	0	0	0	0	0	0	0
Was randomizing appropriate done?	N/A	N/A	N/A	N/A	N/A	N/A	N/A	N/A	N/A	N/A	N/A	N/A	N/A	N/A
5. Outcomes														
Were the outcome measures reliable?	1	0	0	1	1	1	1	1	1	1	1	1	1	1
Were the outcome measures valid?	1	0	0	1	1	1	1	1	1	1	0	1	1	1
6. Intervention														
Intervention was described in detail?	1	1	1	0	1	1	0	1	1	1	1	1	1	1
Contamination was avoided?	N/A	N/A	N/A	N/A	N/A	N/A	N/A	N/A	N/A	N/A	N/A	N/A	N/A	N/A
Cointervention was avoided?	N/A	N/A	N/A	N/A	N/A	N/A	N/A	N/A	N/A	N/A	N/A	N/A	N/A	N/A
7. Results														
Results were reported in terms of statistical significance?	0	1	1	1	1	1	1	1	0	1	0	1	1	1

Were the analysis method/s appropriate?	0	0	1	1	1	1	0	1	1	1	0	1	1	1
Clinical importance was reported?	0	1	1	0	1	1	0	1	1	1	0	0	0	1
Drop-outs were reported?	1	1	1	1	1	1	1	1	1	1	1	0	0	0
8. Conclusion														
Conclusions were appropriate given study methods and results?	0	1	0	1	1	0	0	0	1	0	1	0	0	0
Total	6	9	7	10	11	10	7	10	10	10	5	8	8	9
%	50	75	58	83	92	83	58	83	83	83	42	67	67	75

Yes = 1 point; No = 0 points; CC = Case Control study; CR = Case study; RCT = Randomised Controlled Trial; C = Cohort study; N/A = Not applicable
