# Supplementary material for: Functional outcome of 2-D- and 3-D-guided corrective forearm osteotomies: a systematic review
Source: J Hand Surg Eur Vol. 2023 Sep 25;49(7):843–51. doi: 10.1177/17531934231201962 (PMC11264531; doi:10.1177/17531934231201962)
Supplement: sj-pdf-7-jhs-10.1177_17531934231201962 - Supplemental material for Functional outcome of 2-D- and 3-D-guided corrective forearm osteotomies: a systematic review [file sj-pdf-7-jhs-10.1177_17531934231201962.pdf]

Online Table S6: Quality assessment according to the McMaster score, part four.

Categories	Robinson, 2021	Roner, 2020	Rothenfluh, 2013	Schurko, 2020	Shintani, 2018	Singh, 2022	Stirling, 2020	Tarallo, 2014	Tiren, 2014	Wu, 2017	Zhang, 2022
1. Study purpose											
Was the study question clearly stated?	0	0	0	1	0	1	1	1	0	0	1
2. Literature review											
Was relevant background literature reviewed?	1	1	1	1	0	0	1	0	1	1	0
3. Study design	CS	CS	CS	CS	CS	CS	CS	C	CS	CS	CS
4. Sample											
Was the sample described in detail?	1	1	0	1	1	1	1	0	1	1	1
Was the sample justified?	1	1	1	1	0	1	1	1	1	1	0
Were the groups randomized?	0	0	0	0	0	0	0	0	0	0	0
Was randomizing appropriate done?	N/A	N/A	N/A	N/A	N/A	N/A	N/A	N/A	N/A	N/A	N/A
5. Outcomes											
Were the outcome measures reliable?	0	0	1	1	1	1	0	1	1	1	1
Were the outcome measures valid?	1	0	1	1	1	1	1	1	1	1	1
6. Intervention											
Intervention was described in detail?	1	1	1	1	1	1	1	1	1	1	1
Contamination was avoided?	N/A	N/A	N/A	N/A	N/A	N/A	N/A	N/A	N/A	N/A	N/A
Cointervention was avoided?	N/A	N/A	N/A	N/A	N/A	N/A	N/A	N/A	N/A	N/A	N/A
7. Results											
Results were reported in terms of statistical significance?	0	1	1	1	0	0	1	1	1	1	1
Were the analysis method/s appropriate?	0	0	0	1	1	1	1	1	1	1	1

Yes = 1 point; No =

Clinical importance was reported?	0	0	1	1	0	1	1	1	1	0	1	0
Drop-outs were reported?	1	1	1	1	1	1	1	1	1	1	1	
8. Conclusion												
Conclusions were appropriate given study methods and results?	0	0	0	1	0	0	0	0	1	0	1	
Total	6	6	8	12	6	9	10	9	11	9	10	
%	50	50	67	100	50	75	83	75	92	75	83	

points; CC = Case Control study; CR = Case study; RCT = Randomised Controlled Trial; C = Cohort study; N/A = Not applicable
