# Supplementary material for: Functional outcome of 2-D- and 3-D-guided corrective forearm osteotomies: a systematic review
Source: J Hand Surg Eur Vol. 2023 Sep 25;49(7):843–51. doi: 10.1177/17531934231201962 (PMC11264531; doi:10.1177/17531934231201962)
Supplement: sj-pdf-8-jhs-10.1177_17531934231201962 - Supplemental material for Functional outcome of 2-D- and 3-D-guided corrective forearm osteotomies: a systematic review [file sj-pdf-8-jhs-10.1177_17531934231201962.pdf]

Online Table S7: Different types of complications for conventional and 3D-guided osteotomies.

Complications	Conventional	3D-guided
Implant failure	2.10%	1.97%
Loss of the correction	0.82%	0.00%
A revision corrective osteotomy	1.40%	1.97%
Tendon injuries	2.10%	1.57%
Nerve injuries	0.93%	1.18%
Infection	1.05%	0.00%
Nonunion	2.45%	0.00%
